# Respiratory pathogenic microbial infections: a narrative review

**DOI:** 10.7150/ijms.93628

**Published:** 2024-03-17

**Authors:** Yiyin Long, Yan Zheng, Changlin Li, Zhanjun Guo, Peng Li, Fuqing Zhang, Wei Liu, Yuliang Wang

**Affiliations:** 1 Tianjin Institute of Urology, The Second Hospital of Tianjin Medical University, Tianjin 300211, China.; 2 Department of Radiology, Tianjin First Center Hospital, Tianjin 300192, China.; 3 Department of Neurology, The Second Hospital of Tianjin Medical University, Tianjin 300211, China.; 4 Tianjin Children's Hospital, Children's Hospital, Tianjin University, Tianjin 300134, China.

**Keywords:** Respiratory viruses, Respiratory bacteria, COVID-19, Vaccination

## Abstract

Respiratory infectious diseases have long been recognised as a substantial global healthcare burden and are one of the leading causes of death worldwide, particularly in vulnerable individuals. In the post COVID-19 era, there has been a surge in the prevalence of influenza virus A and other multiple known viruses causing cold compared with during the same period in the previous three years, which coincided with countries easing COVID-19 restrictions worldwide. This article aims to review community-acquired respiratory illnesses covering a broad spectrum of viruses, bacteria, and atypical microorganisms and focuses on the cluster prevalence of multiple known respiratory pathogens in China, thereby providing effective prevention and control measures.

## Introduction

The transmission of respiratory infectious diseases has been reported to have seasonal or epidemic pattern over the last two decades 1. On 23 February, 2023, Chinese authorities announced the end of coronavirus disease 2019 (COVID-19) epidemic, thereby bringing in the first “new normal” year without pandemic in China since the COVID-19 pandemic began in 2020 2. However, surveillance data have indicated an increase in outpatient consultations and hospital admissions of children owing to *Mycoplasma pneumoniae* pneumonia (MPP) since May 2023, and a nationwide surge in the cluster prevalence of multiple known respiratory viral and bacterial pathogens since mid-October, coinciding with a decline in the number of cases of COVID-19 over the same time period. Community-acquired respiratory illness caused by multiple known respiratory pathogens occurs more than one month earlier than historically experienced, which interferes with the timely administration of recommended prophylaxis and influenza vaccination in vulnerable groups, including not only young children but also the elderly 3. Experts have attributed the rise to this phenomenon known as “immunity debt”, which has been observed worldwide after countries eased COVID-19 restrictions 4. We can assume the body's immune system as “a blank sheet of paper”, that is, a population with little-to-no effective cellular immunological memory and responses against respiratory viruses, which have previously caused infection, due to lack of contact and exposure 5. This article aims to review community-acquired respiratory illnesses covering a broad spectrum of viruses, bacteria, and atypical microorganisms, and focuses on the cluster prevalence of multiple known respiratory pathogens in China, thereby providing effective prevention and control measures.

## Global spread of highly pathogenic acute respiratory virus epidemics or pandemics

**Figure 1:**
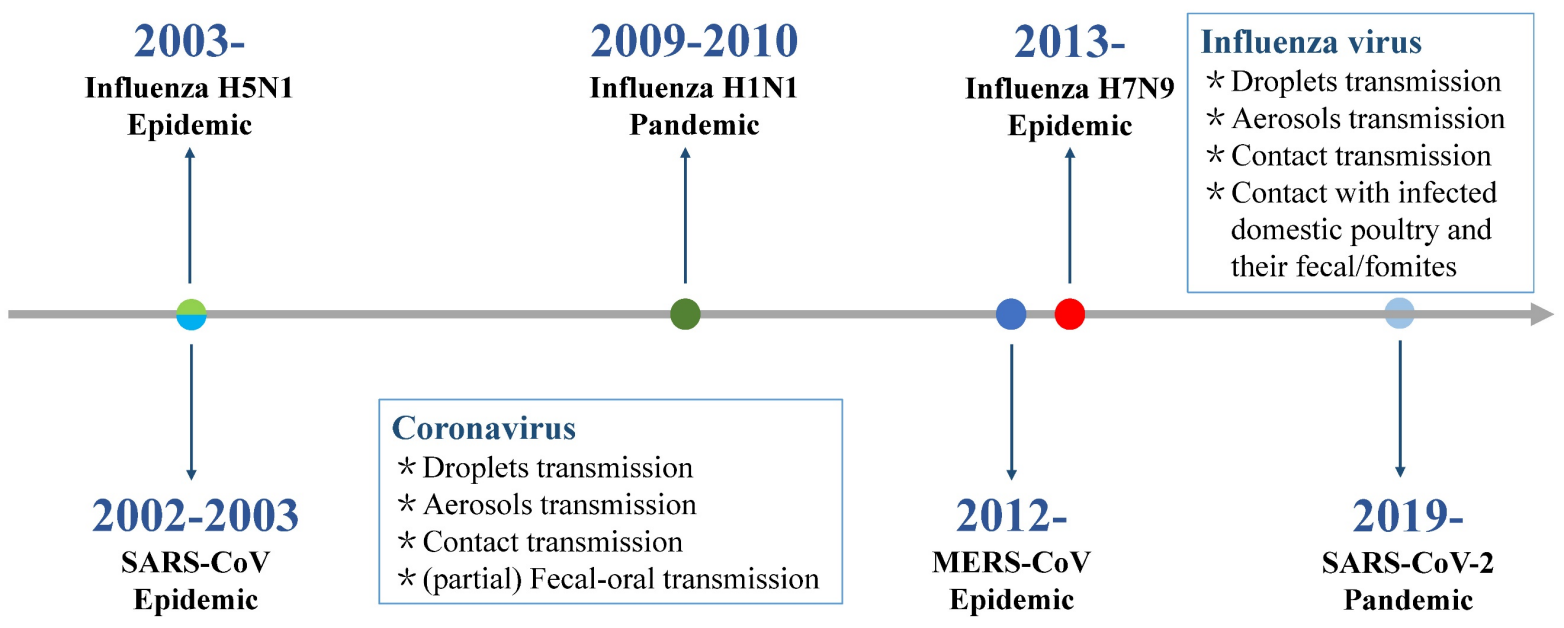
Timeline of epidemics and pandemics caused by highly pathogenic acute respiratory virus.

Within the past 20 years, highly pathogenic acute respiratory viral infections have caused six global epidemics and pandemics with high morbidity and mortality rates (**Figure 1**). These were caused by respiratory RNA viruses with typical characteristics of effective transmission and high mutation rate, and includes the original severe acute respiratory syndrome coronavirus (SARS-CoV) infection causing acute respiratory distress syndrome and multiple organ dysfunction syndrome in China (2002) 6, highly pathogenic avian influenza virus H5N1 infection causing severe disease with high mortality in humans in parts of Asia (mid-December 2003) 7, the swine influenza virus A H1N1 infection causing mild disease in most cases in 2009 (five continents) 8, the Middle East respiratory syndrome coronavirus (MERS-CoV) infection 9 that caused death of approximately 36% of patients with MERS in Saudi Arabia (2012), novel avian influenza virus H7N9 causing severe acute respiratory infection in China (2013) 10, and the current circulating severe acute respiratory syndrome coronavirus 2 (SARS-CoV-2) infection. SARS-CoV-2 causing COVID-19 was first identified in December 2019 in Wuhan, China, has spread as a global pandemic, and in March 2020, it was declared a “public health emergency of international concern” by the World Health Organization 11.

## Respiratory pathogenic microorganisms

### Respiratory pathogenic viruses

Acute respiratory diseases caused by various types of viruses have been identified as a global public health threat and are one of the leading causes of death among vulnerable individuals 12. Respiratory viruses can be classified based on their nucleic acid composition: RNA viruses and DNA viruses. RNA viruses include influenza viruses A and B 13, parainfluenza virus (PIV) 14, human metapneumovirus (hMPV) 15, respiratory syncytial virus (RSV; now known as human orthopneumovirus) 16-18, coronaviruses 19-21, and human rhinovirus (HRV) 22. DNA viruses implicated in respiratory infections include adenoviruses (Adv) 23,24 and human bocavirus (HBoV) type 1 25. It is worth mentioning that over the past two decades, respiratory infection epidemics have predominantly been caused by RNA viruses. Consequently, the development of effective therapeutics targeting respiratory RNA viruses is imperative in the battle against infectious diseases. The families and genera of common respiratory viruses are summarised in Table 1. The clinical presentation of respiratory viral infections can range from mild upper respiratory and/or gastrointestinal symptoms to more severe tracheobronchitis and pneumonia.

### Respiratory pathogenic bacteria and atypical microorganisms

The main pathogenic gram-positive bacteria responsible for respiratory ailments include *Streptococcus pneumoniae* 26, *Group A Streptococcus* 27, *Streptococcus agalactiae* 28, *Staphylococcus aureus* 29, *Mycobacterium tuberculosis* 30, and *Corynebacterium* diphtheriae 31. The main pathogenic Gram-negative bacteria implicated are *Bordetella parapertussis*, *Bordetella pertussis*, *Bordetella bronchiseptica* 32,33, *Haemophilus influenzae type b* 34,35, *Klebsiella pneumoniae* 36, *Acinetobacter baumannii* 37, *Pseudomonas aeruginosa* 38,39, and Legionella pneumophila 40,41, which are associated with antimicrobial resistance, posing the greatest threat to modern public health 42.

Based on the Global Burden of Diseases, Injuries, and Risk Factors Study (GBD) 2019 data 43, the pathogenic bacterium *S. pneumoniae* was the leading cause of fatal lower respiratory infections with 653 000 deaths and associated with the most deaths among children younger than 5 years. *Chlamydophila pneumoniae* 44 and *M. pneumoniae* (MP) 45 are common pathogens responsible for community-acquired pneumonia in children. The families and genera of respiratory pathogenic bacteria and atypical microorganisms are summarised in Table 1.

### Respiratory pathogen co-infections

When two or more respiratory pathogens are clinically observed to co-circulate in a region, co-infection inevitably emerges, and their synergistic or superimposed pathogenicity, in many cases, likely enhances the severity of community-acquired pneumonia. For example, M Abd El-Halim et al. 46 studied post-COVID-19 lower respiratory tract co-infections and showed that SARS-COV-2 respiratory co-infections were mainly caused by bacterial pathogens, and most commonly *Klebsiella species* (spp.). Most bacterial co-infections are caused by multidrug-resistant strains. Ruttoh et al. 47 implemented an active sentinel surveillance system to detect SARS-CoV-2 and co-infections with other acute respiratory pathogens (12 viruses and seven bacteria), and approximately a third of SARS-CoV-2 positive individuals were co-infected with one or more acute respiratory pathogens. The most common co-infecting pathogens were *S. pneumoniae*, *H. influenzae*, and human coronavirus OC43 (HCoV-OC43). In two cases, four pathogens (human coronavirus 229E, HCoV-OC43, *H. influenzae*, and *S. pneumoniae*) were coinfected with SARS-CoV-2. Sato et al. 48 estimated the clinical and virological impact of influenza and other respiratory virus co-infections in children, and suggested that it was necessary to assess clinical symptoms as well as the levels of detected viruses to determine which virus contributed to the development of illness when multiple respiratory viruses were detected in the same patient. Conversely, Weidmann et al. 49 reviewed the co-infection rates of respiratory viruses in patients presenting respiratory symptoms who visited their medical centre in New York City, and suggested a viral exclusionary effect between most seasonal respiratory viruses, including SARS-CoV-2, influenza virus, and RSV. They also demonstrated a significant burden of respiratory viral co-infections among children.

## Current circulating respiratory pathogens in China

### Epidemiological characteristics

**Figure 2:**
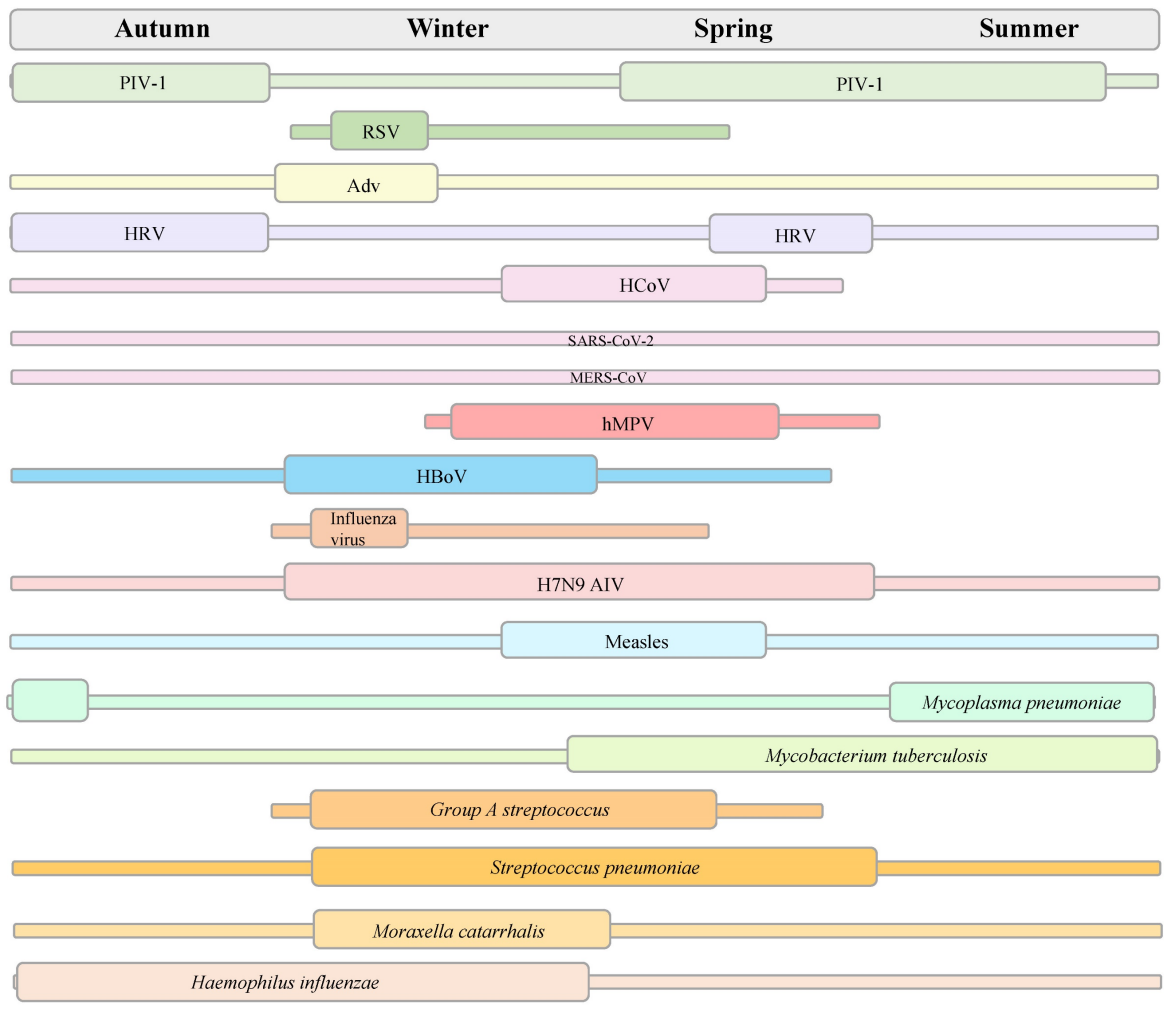
Seasonality trends of some respiratory pathogens. RSV: respiratory syncytial virus; PIV: parainfluenza virus; Adv: adenovirus; HRV: human rhinovirus; HCoV: human coronaviruses; SARS: severe acute respiratory syndromes; MERS: Middle East respiratory syndrome; hMPV: human metapneumovirus; HboV-1: human bocavirus; AIV: avian influenza virus.

Respiratory infection management should consider investigating the most likely pathogens based on syndromic surveillance data from the host country. China has established the Chinese National Influenza Center to monitor and control the influenza epidemic. The national influenza test positivity rate (the number of positive samples/number of test samples × 100%) increased between the 45th week of 2018 (as of 27 November 2018) and the 13th week of 2019 (as of 31 March 2019) and peaked at 32.7%, while the influenza activity in summer and fall remained at a low level of 2% 50. These results indicate that respiratory viral infections tend to follow seasonal patterns, with a high incidence in dry and cold winters, particularly in temperate regions 51-55 (**Figure 2**). In addition to the seasonal cycle, the main epidemiological characteristics of respiratory infectious diseases include diverse respiratory pathogens and clinical manifestations, high contagion potential and transmissibility, global pandemic potential, and vaccine prevention for only partial respiratory infections. In fact, the epidemiological surveillance of respiratory diseases during the COVID-19 pandemic and after the relaxation of restrictions also showed a consistent epidemic pattern 56. However, their transmission dynamics were different, which were marked by “off-season” pattern during COVID-19 pandemic. Implementing control measures during COVID-19 contributed to the low incidence of various infectious diseases, including measles, pertussis, scarlet fever, seasonal influenza, mumps, and rubella, which showed a more than 50% reduction in the prevalence of these disease compared to 2019 57,58. For example, the number of seasonal influenza cases decreased from 3,507,306 in 2019 to 1,881,460 in 2020-2021, and influenza activity decreased by approximately 79% 58,59.

By contrast, current transmission dynamics of respiratory diseases were marked by “upsurge” pattern. In fact, the observed “off-season and "upsurge” patterns can be attributed to the change of public health mitigation measures for COVID-19 60. According to the Chinese authorities from the National Health Commission, the current reemergence and surging number of respiratory diseases, which continue to contribute to the clinical burden, have been fuelled by the easing COVID-19 restrictions and arrival of the cold season 61. Seasonal influenza started to spread in communities in China from 2023 spring, and the number of cases exceeded the pre-pandemic and pandemic numbers, with an estimated infection attack rate of 28.30% in southern China and 18.51% in northern China 62. In addition, northern China has reported an upsurge in the clusters of respiratory illnesses in children since mid-October compared to the same period in the previous three years 63. A higher number of children are affected, partly because the current circulating pathogens included *MP and* RSV, which are known to affect children to a greater extent than that of adults. According to surveillance results, the currently prevalent respiratory cases in China are due to known respiratory pathogens, such as influenza virus A H3N2, Adv, hMPV, HRV, SARS-CoV-2, PIV, *H. influenzae*, and *S. pneumoniae*. Some cases of respiratory infections by other pathogens, including *B. pertussis* and *C. pneumoniae*, have also been reported 55. These pathogens infect the cells of the respiratory tract, causing different illnesses ranging from the common cold to severe pneumonia, with different clinical characteristics 64-75 (Table 2). In addition, differences in the prevalence patterns of respiratory pathogens were observed among different age groups 76 (Table 3). The 5-14 year age group had a higher prevalence of infection with Mycoplasma and Adv, while those 60 years and older had a higher prevalence of infection with HMPV and coronavirus than the other age groups. Moreover, compared with the 1-4 year age group, those 15-59 years and older had a higher prevalence of SARS-CoV-2 infection. It is worth emphasising that case reports of co-infections or superimposed infections between virus-virus, bacterium-bacterium, or virus-bacterium have increased in the short term. This is because the number of cases of some of these diseases increased significantly during the same period, and the main clinical epidemiological characteristics of circulating respiratory pathogens included co-infections, superimposed infections, or repeat infections. Therefore, we highlight the need for continued vigilance and education regarding the recovery and necessary precautions following infection.

### *In vitro* diagnosis

**Figure 3:**
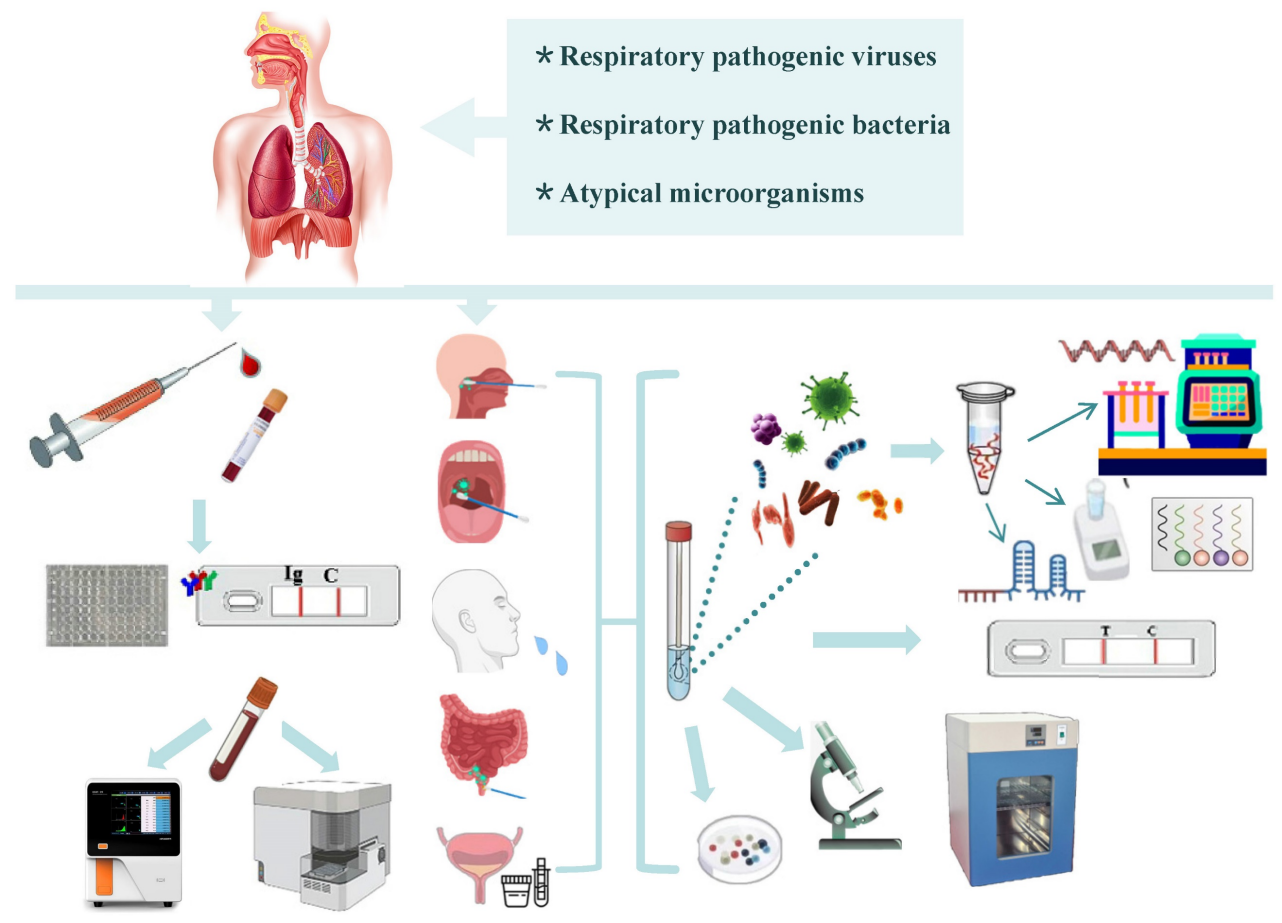
*In vitro* diagnosis for respiratory pathogenic microorganisms.

As clinical manifestations may be similar among respiratory viral infections and early-stage discrimination between respiratory viral and bacterial infections is essential, accurate yet rapid and accessible *in vitro* diagnosis (IVD) is needed for circulating respiratory pathogens to reduce inappropriate prescriptions and antibiotic resistance and improve outcomes. Multiple clinical specimen types for non-invasive detection include the upper respiratory tract (nasopharyngeal, oropharyngeal, and saliva), lower respiratory tract (deep cough sputum and bronchoalveolar lavage fluid), digestive tract (anal swab), and urinary tract (urine) 77. A range of laboratory-based non-invasive diagnostic tools are available as follows: rapid point-of-care tests (POCT) (rapid antigen detection, loop-mediated isothermal amplification (LAMP) assays, recombinase polymerase amplification assays, microfluidic chips) 78, standard analysis (serum immunoassay using antibodies, urine antigen detection, immunochemistry, smear microscopy, blood/sputum cultures, electron microscopy, mass spectrometry assay, and molecular diagnostics) 79,80, and auxiliary detection (whole blood cells counts, procalcitonin, C-reactive protein, D-dimer, T-cell activation-induced marker assays) 81,82. Molecular diagnostics is considered the gold standard for viral diagnosis 83. A range of molecular diagnostic techniques available include real-time polymerase chain reaction (PCR), real-time reverse transcription PCR (RT-PCR), multiplex RT-PCR, digital PCR, LAMP, clustered regularly interspaced short palindromic repeats (CRISPRs), nucleic acid microarray, real-time metagenomics, and next-generation sequencing (NGS). **Figure 3** shows i*n vitro* diagnosis tests for the detection of pathogenic respiratory microbes. It is necessary to optimise the detection indicators according to the clinical manifestations induced by different pathogens in patients of all ages to identify infections and prevent missed diagnoses.

### Radiological diagnosis

**Figure 4:**
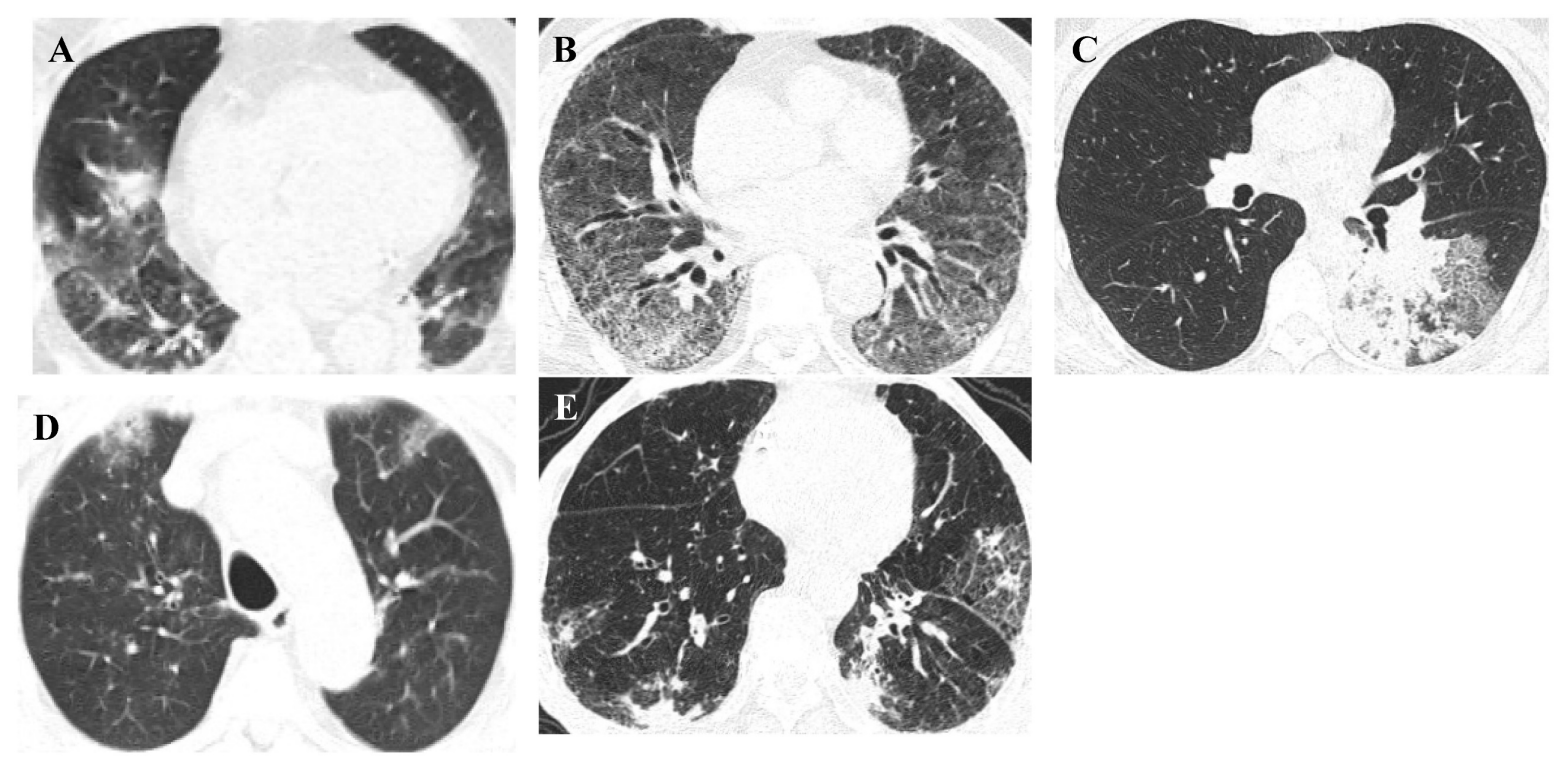
Representative HRCT-scan images of pulmonary lesions. Representative (**A**) adenovirus pneumonia, HRCT: Both lungs show patchy consolidation with ground-glass opacities, (**B**) respiratory syncytial virus pneumonia, HRCT: Both lungs show ground-glass opacities and reticular high-density shadows, (**C**) *Mycoplasma pneumoniae* pneumonia, HRCT: The lower lobe of the left lung shows patchy high-density shadows and reticular high-density shadows, (**D**) COVID-19 pneumonia, HRCT: Both lungs show subpleural patchy ground-glass opacities, (**E**) influenza virus A pneumonia, HRCT: Both lungs show patchy high-density shadows and reticular high-density shadows under the pleura.

Radiological diagnosis is essential for the evaluation of suspected or confirmed infectious patients with an initial diagnosis, evaluation of disease progression and complications, and monitoring the response for appropriate disease management 84. Different respiratory infections cause abnormal symptoms in the lung parenchyma (internal structures, texture, and density) that can be seen on conventional imaging modalities, such as chest computed tomography (CT). Furthermore, developing a deep learning algorithm, Pneumonia-Plus, based on CT images may be utilised to screen and differentiate between bacterial, fungal, and viral pneumonia, which reduces the risk of misdiagnosis and is important for appropriate treatment, avoiding the use of unnecessary antibiotics and providing timely information to guide clinical decision-making and improve patient outcomes 85,86. Representative high-resolution CT (HRCT) scan images of pulmonary lesions with respiratory pathogenic microbes are shown in **Figure 4**.

### Why immunological protection is important

Currently, four terms explain the surge in respiratory illness in the post-pandemic era. One is called as “immunity debt”, which refers to a long-term lack of immune memory against a given respiratory pathogen leading to loss of immunological protection/defence against re-exposures to the same pathogen due to strict COVID precautions, which can make people experience more intense symptoms of infection 87 and has negative consequences owing to their increased circulation and transmission in the community when unpredictable epidemics occur 88. Indeed, during the post-COVID period, an unusually sharp increase and earlier peak of community-acquired respiratory illnesses has entailed an overload of hospital outpatients, emergencies, and wards, which exerts substantial pressure on hospital administration 89. The second descriptive term is called as “immunity gap” that was also invented after the pandemic. This means that a dramatic reduction in the circulation of other respiratory pathogens leads to a cohort of children with few immunological defence mechanisms against bugs such as influenza, RSV, and other viruses causing cold, setting the stage for large outbreaks when these pathogens return 88. An American emergency physician and public health expert, used the expression “immune naive” to explain that there is a cohort of young children who are never exposed to a bunch of seasonal pathogens for a few years and are therefore “immune naive” to them 90. Together, we metaphorize the body's immune system as “a blank sheet of paper” (a rhetorical device) for the population with little-to-no primary response against respiratory viruses due to strict COVID precautions. Pan et al. 57 suggested that the widespread and lasting immune dysfunction caused by SARS-CoV-2 may be the reason for the resurgence of influenza virus. Some scientists call this phenomenon “immunity theft”, which refers to the notion that SARS-CoV-2 itself steals immunity, making people who had COVID-19 more susceptible to other infections 91. All persons aged 6 months or older are strongly recommended polyvalent influenza vaccination using a one-dose plus mass catch-up strategy and equitable immunisation coverage, being the most cost-effective method 92.

### Vaccination

Vaccination is essential during pandemics. Immune responses to pathogenic microbes can be established in two ways: natural infection and vaccination. Under the insufficiency of natural infection, vaccination is the best way to prevent infection with community-acquired respiratory pathogen or spreading it to other people, leading to repay “immune debt” so that the immune system of susceptible body is no longer a blank sheet of paper 60. Extensive vaccination is safe and efficient and helps people fight the infection 93; however, multiple doses and booster doses are needed to produce and sustain protective immunity to dramatically decrease the infection rate, severity, and mortality in high-risk groups 94. Furthermore, it is important to sustain high vaccination coverage and further enhance the National Immunization Program, particularly for older adults, children, immunocompromised individuals, and healthcare workers having a high risk of exposure 95. Currently available different types of vaccines include protein subunit, inactivated, live‐attenuated, and virus-like particle vaccines, recombinant fusion protein vaccines, recombinant adenovirus vaccines, mRNA vaccines, unmodified RNA-based vaccines, and nano vaccines 96,97. Studies have confirmed that the optimal protective response differs according to the vaccine formulation and delivery. Moscara et al. showed that the simultaneous administration of anti-SARS-CoV-2 and seasonal influenza vaccines should be encouraged to increase vaccination coverage 98. Furthermore, intramuscular injection is a major route of vaccination that induces systemic cellular immune memory and humoral immunity 99. Alternatively, as a subgroup of the mucosal delivery system, intranasal vaccination may induce both localised mucosal protection and systemic immune responses by boosting mucosal immunoglobulins and cellular immune responses in the respiratory tract 100. In the current respiratory disease epidemic in China, it is recommended that people over 6 months of age without contraindications actively vaccinate related vaccines as they can get mainly according to the characteristics of vaccines, disease prevention and control needs to decide, especially “one old and one young”, and the main types of vaccines to prevent respiratory diseases include inactivated/live-attenuated influenza virus vaccine, COVID-19 vaccine, pneumococcal polysaccharide vaccine, and haemophilus influenzae type b vaccine. Driving National Immunization Program needs to consider factors including immunisation strategies, financial budget, immunisation service costs, and social supply- and demand-side factors 92.

## Conclusions and Perspectives

The management of acute respiratory infections should consider the investigation and empirical coverage of the most likely agents based on syndromic surveillance data from the host country and/or other relevant exposure histories during events. Given the severity of the situation in which public health measures, healthcare administrations/systems, and economics are challenged due to increased incidence and related hospitalisations of multiple known respiratory diseases, it is critical to follow multiple measures to block the spread of infectious disease pathogens. These include good hygiene practices of individuals, annual receiving of recommended vaccination and adequate coverage, rapid detection and diagnosis of the early stage of infection and timely medical care as needed, keeping distance from people who are ill, wearing masks in cluster places, ensuring good ventilation, multiple micronutrient supplementation, and supporting psychological health and well-being 101,103. Nevertheless, breakthrough infections remain a challenge for disease control and attenuation of disease severity 103. An update on SARS-CoV-2 new variant JN.1 shows that the new variant has been detected and has triggered a new wave of infections in 40 countries around the world and may become more common relative to other circulating variants. Centers for Disease Control and Prevention (CDC) projects that the JN.1 comprises an estimated 15-29% of the currently circulating variants in the United States as of 8 December, 2023 104. Furthermore, seven cases of JN.1 were reported in China as of 10 December 2023, according to the National Disease Control and Prevention Administration. Notably, on 20 December 2023, the UN World Health Organization designated the JN.1 'variant of interest' amid sharp rise in global spread 105. Thus, an updated COVID-19 vaccine could protect against the JN.1 and other variants. A further increase in the preparation and development of vaccines (via plug-and-play technical platforms) and antivirals or antibiotics (avoiding multiple resistance) and optimisation of laboratory diagnostic techniques will hopefully help deal with persistent community-acquired respiratory illnesses and potential viral contagious diseases that might arise in the future, that is, a grey rhino. As a tool in potential uses for implementing early warning and tracking capabilities, further research is required to use advanced algorithms with artificial intelligence (AI) and machine learning capabilities to diagnose and predict respiratory illnesses 106. Overall, the transmissibility, prevalence, and epidemic patterns of seasonal respiratory viruses are dynamic with year-to-year heterogeneity, and it is a high time to get the vaccine during this fall if you have not received one. The next “Disease X” could appear at any time, any regions and any population 107,108, the world needs to be better prepared for the enhanced disease surveillance in healthcare facilities and community settings, along with strengthening the capacity of the health system to manage patients.

## Figures and Tables

**Table 1:** Respiratory pathogenic microorganisms

Type	Family	Genus	Species	Refs.
**Respiratory viruses**				
RNA viruses	Orthomyxoviridae	*Influenza A*	All influenza A subtypes	13
		*Influenza B*	Influenza B	
	Paramyxoviridae	*Rubulavirus*	PIV-2	14
			PIV-4a	
			PIV-4b	
		*Respirovirus*	PIV-1	
			PIV-3	
	Pneumoviridae	*Metapneumovirus*	hMPV	15
		*Orthopneumovirus*	RSV-A	16-18
			RSA-B	
	Coronaviridae	*Betacoronavirus*	SARS-CoV	19
			MERS-CoV	
			SARS‐CoV‐2	
			HCoV-NL63	20,21
			HCoV‐229E	
			HCoV‐OC43	
			HCoV-HKU1	
	Picornaviridae	*Enterovirus*	HRV A,B,C	22
DNA viruses	Adenoviridae	*Mastadenovirus*	Adv	23,24
	Parvoviridae	*Bocavirus*	HBoV- 1	25
**Respiratory bacteria**				
Gram-positive bacteria	Streptococcaceae	*Streptococcus*	*Streptococcus pneumoniae*	26
			*Group A streptococcus*	27
			*Streptococcus agalactiae*	28
	Staphylococcaceae	*Staphylococcus*	*Staphylococcus aureus*	29
	Mycobacteriaceae	*Mycobacterium*	*Mycobacterium tuberculosis*	30
	Corynebacteriaceae	*Corynebacterium*	*Corynebacterium diphtheriae*	31
Gram-negative bacteria	Bogoriellaceae	*Bordetella*	*Bordetella parapertussis*	32,33
			*Bordetella pertussis*	
			*Bordetella bronchiseptica*	
	Pasteurellaceae	*Haemophilus*	*Haemophilus influenzae type b*	34,35
	Enterobacteriaceae	*Klebsiella*	*Klebsiella pneumoniae*	36
	Moraxellaceae	*Acinetobacter*	*Acinetobacter baumannii*	37
	Pseudomonadaceae	*Pseudomonas*	*Pseudomonas aeruginosa*	38,39
	Legionellaceae	*Legionella*	*Legionella pneumophila*	40,41
**Atypical microorganisms**				
Chlamydia	Chlamydiaceae	*Chlamydia*	*Chlamydophila pneumoniae*	44
Mycoplasma	Mycoplasmataceae	*Mycoplasma*	*Mycoplasma pneumoniae*	45

PIV: parainfluenza virus; hMPV: human metapneumovirus; RSV: respiratory syncytial virus; SARS: severe acute respiratory syndromes; MERS: Middle East respiratory syndrome; HCoV: human coronaviruses; HRV: human rhinovirus; Adv: adenovirus; HboV-1: human bocavirus

**Table 2:** Comparison of clinical characteristics and treatment between major prevalent respiratory pathogens in China

Pathogens	Incubation period	Signs and Symptoms	Self-limiting	People at increased risk	Treatment	Refs.
Influenza virus A H3N2	1-4 days	- Characterization: acute onset of fever, muscle and joint pain, headache, malaise, dry cough, sore throat, runny nose/nasal congestion, and severe malaise (fatigue) - Gastrointestinal symptoms: nausea, vomiting and diarrhoea	- Self-limiting: recovery wthin 5-7 days	- People of all ages	- Antiviral therapy (Oseltamivir)	64
MP	1-4 weeks	- Commonly mild symptoms: tracheobronchitis: sore throat, feeling tired, fever slowly, worsening, cough, and headache - Sometimes pneumonia: fever and chills, cough, feeling tired, and shortness of breath	- Self-limiting: recovery in a week to three	- People of all ages (most commonly seen in young adults and school-aged children)	- Antibiotics	65
AdV	3-5 days	- Common cold or flu-like symptoms: fever, sore throat, acute bronchitis, pneumonia, pink eye, and acute gastroenteritis	- Self-limiting: recovery within a week	- People of all ages (most commonly seen in children younger than 5 years old)	- Symptoms relief	66
HMPV	3-6 days	- Cough, fever, nasal congestion, and shortness of breath - Bronchitis - Pneumonia	- Self-limiting: recovery within a week	- People of all ages	- Supportive care - Symptoms relief	67
RSV	4-6 days	- Runny nose, decrease in appetite, coughing, sneezing, fever, and wheezing - Very young infants: irritability, decreased activity, and breathing difficulties	- Self-limiting: recovery in a week or two	- People of all ages (most commonly seen in children younger than 2 years old)	- Symptoms relief - Multiple, new RSV immunizations	68
HRV	2-5 days	- Many people will have no or mild symptoms: cough, sneeze, runny nose, nasal congestion, sore throat, headache, body aches, and fever - More severe illness is less common: asthma exacerbations, bronchioliti, middle ear infections, sinusitis, bronchitis, or pneumonia	- Self-limiting: recovery in a week or two	- People of all ages	- Symptoms relief	69
SARS-CoV-2	2-14 days	- Fever and dry cough - Acute pneumonia-associated symptoms	- Self-limiting	- People of all ages	-Symptoms relief - Antiviral treatments - Immunizations	70,71
PIVs	2-6 days	- Fever, runny nose, cough, sneezing, and sore throat - Croup - Pneumonia	- Self-limiting	- People of all ages (most commonly seen in infants and young children)	- Symptoms relief	72
*Haemophilus influenzae*	12-48 h	- Pneumonia: fever and chills, cough, shortness of breath or difficulty breathing, sweating, chest pain, headache, muscle pain or aches, excessive tiredness - Bloodstream infection: fever and chills, excessive tiredness, pain in the belly, nausea with or without vomiting, diarrhea, anxiety, shortness of breath or difficulty breathing, and altered mental status (confusion)		- Children younger than 5 years old and adults 65 years and older	- Antibiotics - Symptoms relief	73
*Streptococcus pneumoniae*	1-3 weeks	- Dyspnea, cough, pleuritic pain, sputum production, and fever - Pneumonia - Meningitis - Febrile bacteraemia - Otitis media - Sinusitis - Bronchitis		- People of all ages	- Antibiotic therapy - Supportive care - Pneumococcal conjugate vaccine (PCV)	74,75

MP: *Mycoplasma pneumoniae*; Adv: adenovirus; hMPV: human metapneumovirus; RSV: respiratory syncytial virus; HRV: human rhinovirus; SARS: severe acute respiratory syndromes; PIV: parainfluenza virus

**Table 3:** Major respiratory pathogens prevalent in different age groups in China

Age group (years)	Major prevalent pathogens
1-4	Influenza virus, rhinovirus
5-14	Influenza virus, mycoplasma, adenovirus
15-59	Influenza virus, rhinovirus, SARS-CoV-2
≥60	Influenza virus, human metapneumovirus, coronavirus
